# Assessment Tool of Bipolar Disorder for Primary Health Care: The SAEBD

**DOI:** 10.3390/ijerph18168318

**Published:** 2021-08-06

**Authors:** Jose Manuel Montes, Ana Pascual, Sandra Molins Pascual, Carmen Loeck, Maria Belen Gutiérrez Bermejo, Cristina Jenaro

**Affiliations:** 1 Psychiatry Service, Hospital Ramón y Cajal, 28034 Madrid, Spain; carmen.loeck@gmail.com; 2 Centro de Investigación Biomédica en Red de Salud Mental (CIBERSAM), 28034 Madrid, Spain; 3 Instituto Ramón y Cajal de Investigación Sanitaria (IRYCIS), 28034 Madrid, Spain; 4 Division of Psychiatry, Imperial College London, London W12 0NN, UK; anapascualsan@gmail.com; 5 Institute of Legal Medicine of Valencia, 46013 Valencia, Spain; molins_sanpas@gva.es; 6 Protección de las Personas con Discapacidad (PROTEDIS), Faculty of Psychology, Universidad Nacional de Educación a Distancia (UNED), 28040 Madrid, Spain; mbgutierrez@psi.uned.es; 7 Instituto Universitario de Integración en la Comunidad (INICO), Faculty of Psychology, Universidad de Salamanca, 37005 Salamanca, Spain

**Keywords:** bipolar disorder, assessment, primary health care, receiver-operating-characteristics curve, sensitivity and specificity, rating scales, SAEBD

## Abstract

Mixed states are highly prevalent in patients with bipolar disorder and require comprehensive scales. Considering this, the current study aims to develop a measure to assess the full spectrum of clinical manifestations of bipolar disorder. A sample of 88 patients was evaluated; the Hamilton Depression Scale (HAM-D), Montgomery-Asberg Depression Scale (MADRS), and the Young Mania Rating Scale (YMRS) were applied, together with the preliminary version of the Scale for the Assessment of Episodes in Bipolar Disorder (SAEBD). After analyzing the appropriateness and statistical properties of the items, discriminant analysis and analysis of diagnostic capacity were performed. The discriminant functions correctly classified 100% of the cases in euthymia, predominant depressive symptoms or mixed symptoms, as well as 92.3% of the cases with predominant manic symptoms. Overall, the functions correctly classified 98.9% of the cases. The area under the curve (0.935) showed high capacity to discriminate between clinical and non-clinical cases (i.e., in euthymia). The SAEBD sensitivity was 0.95, specificity was 0.71, the Positive Predictive Value (PPV) was 0.88, the Negative Predictive Value (NPV) was 0.87, the Positive Likelihood Ratio (+LR) was 3.33, and the Negative Likelihood Ratio (−LR) was 0.07. In conclusion, the SAEBD is a promising scale that shows high reliability and validity, as well as diagnostic utility as a screening tool for use in diverse health care settings.

## 1. Introduction

Bipolar Disorder (BD) affects about three percent of the population of all races and cultures and has a major socio-economic impact, both directly through its impact on health care, and indirectly through the loss of productivity of the sufferer [1,2,3]. It is a disorder that is often diagnosed late. A diagnosis could take as long as 10 years, but the mean delay is estimated to be around 6.5 years [4,5,6]. It has been suggested that the delay is shorter if non-specialist mental health professionals can detect symptoms before specialist teams assessed them [7]. Older age and having other mental health symptoms tend to increase diagnostic delay [7]. This delay may lead to untreated bipolar disorders, with potential consequences such as substance abuse, higher risk of hospitalizations and impairment on work or social functioning [8,9]. It is characterized by the presence of depressive and manic symptoms, both during the affective episodes in the acute phases, as well as during their evolution at the subclinical level. The simultaneous presence of symptoms of both polarities is called mixed states and their existence was one of the most convincing arguments put forward by Kraepelin in favor of the unified concept of manic-depressive psychosis [10]. Subsequently, however, mixed states have been relegated, both conceptually and in terms of their treatment, in respect to manic or depressive episodes. This has been partly due to their difficulty in being recognized in the practice and therefore correctly diagnosed. Under-diagnosis and delayed detection of bipolar disorders have been reported in several contexts [11].

The lack of homogeneity in the naming of mixed states in the literature has not contributed to deepening our knowledge of them. The conceptualization of mixed states in the Diagnostic and Statistical Manual of Mental Disorders, fourth edition, text revision (DSM-IV-TR) [12], requiring the simultaneous presence of complete criteria for both depressive and manic episodes simultaneously, was of little value both clinically and in research. Thus, in the Diagnostic and Statistical Manual of Mental Disorders, fifth edition (DSM-5) [13], mixed states have been changed from being considered as categorical episodes to specifiers that can be applied to any affective episode, whether depressive, manic, or hypomanic, or within bipolar disorder as unipolar. However, typical diagnostic tools are usually focused on either manic or depressive symptoms. This may lead to underestimating mixed symptoms in clinical and research practice, with potential consequences (e.g., inappropriate treatment).

The prevalence of mixed states is high and may occur in 30–40% of patients with bipolar disorder throughout the progression of the illness [14]. In a study conducted in 76 centers in Spain, the prevalence of mixed states among admitted patients with bipolar disorder ranged from 9% to 23%, depending on the diagnostic criteria used [15]. Although less recognized, depression with mixed symptoms is also frequent and up to two thirds of patients with depressive episodes may have concurrent manic symptoms [16]. In a study carried out in Canada, a total of 26.0%, 34.0%, and 33.8% of individuals met criteria for Mixed features specifier during an index major depressive episode, as part of major depressive disorder, BD-I and BD-II, respectively [17].

The clinical evolution of patients who experience mixed symptoms will be worse overall than in other patients with bipolar disorder: higher number of relapses, substance abuse, higher number of suicide attempts, higher risk of rapid cycling, comorbidities, longer duration of episodes, worse response to treatments and shorter duration of clinical remission periods [18,19,20,21]. In addition, measures of quality of life and physical health are also worse for patients with mixed symptoms [19].

At the same time, there is little scientific evidence regarding how mixed states should be treated. We know that mixed states have traditionally been associated with a worse and more variable response to treatment [22]. In addition, given the difficulty and poor response to mood stabilizers in mixed states, the possibility of using, and therefore evaluating, alternative treatments such as antipsychotics, in monotherapy or in combination with them, must be taken into account [23]. It is therefore essential to be able to count on adequate assessment instruments for the global evaluation of bipolar disorder, of all episodes, including those with the mixed symptom specifier, to allow their detection and to be used in studies assessing the efficacy of treatments on this group of symptoms. Primary care settings are key in assessing affective symptoms, so brief screening tools that are easily administered as well as comprehensive are a real need. Indeed, according to the National Institute for Health and Care Excellence (NICE) guidelines [24], primary care clinicians should be aware of how to manage bipolar disorder patients in order to decide if a referral is needed to mental health services or not. While waiting for specialist input, it could be helpful for primary healthcare to be aware of manic, depressive, and mixed symptoms in order to monitor symptoms or manage risk (e.g., if an urgent referral is needed to consider an admission).

Currently, there are scales to evaluate the symptoms of mania, the most widely used being the Young Mania Rating Scale (YMRS) [25]. The scale consists of 11 items to be scored by the clinician based on their observations and the patient’s account. It is performed in relation to the clinical history of the last 48 h. The Spanish version is validated by Colom and collaborators [26], and is sensitive to change in clinical trials; however, it is not as useful for the evaluation of symptomatology during hypomanic episodes or possible mixed symptoms during a depressive episode [27].

As mentioned, the DSM-5 considers mixed states as specifiers that can be applied to manic, hypo-manic, or depressive episodes. It is important to point out that the mixed features associated with an episode of unipolar major depression constitute a significant risk factor for the development of bipolar disorder [28] hence, the importance of detecting mixed symptoms and their correct therapeutic approach in this subgroup of patients as well. It is for this reason that the complete evaluation of mixed symptoms in a depressive episode is fundamental. Scales, such as the Hamilton Depression Scale (HAM-D) [29,30], are available for the evaluation of depressive symptoms. This scale consists of 17 items, applied by a clinician, that assess the symptomatological profile and severity of the depressive episode. The reference time frame is the time of the interview, except for certain items. A Spanish version has been validated by Ramos-Brieva [31]. Another instrument for the assessment of depressive symptoms is the Montgomery-Asberg Depression Scale (MADRS) [32], administered by a clinician, it consists of 10 items that assess the symptomatological profile and severity of depression. The score for each item ranges from 0 to 6. The validation of the Spanish version was done by Lobo et al. [33].

Consequently, despite the need for scales that assess and measure the symptoms that occur in bipolar disorder in a comprehensive manner, including mixed symptoms to date, the simultaneous use of one scale for the assessment of mania and another scale for the assessment of depression has been the most commonly used strategy.

Nevertheless, some attempts have been made to design an instrument that could assess mixed symptoms. For example, Cassidy et al. [34] developed the Scale for Manic States based on a mania scale that included both pure mania (15 items) and mixed mania symptoms (5 items). These authors conducted their study with patients with pure or mixed acute mania, without including patients with depression and mixed symptoms. Cavanagh et al. [35] developed a self-applied scale conceived as a bivariate scale with the presence of symptoms of both manic and depressive polarity. In this instrument, each item was divided into “depressive” and “manic” aspects that were assessed relative to zero as an intermediate value. Zimmerman et al. [36] developed another instrument based on the Clinically Useful Depression Outcome Scale (CUDOS). The authors added items to this scale corresponding to the mixed symptom specifiers according to the DSM-5, thus constructing the CUDOS-M. The results of this study are yet to be replicated and validated with larger clinical samples, including patients with bipolar disorder of different characteristics.

Another alternative for evaluating mixed symptoms in patients with depression involves the use of a comprehensive scale, such as the HAM-D, for the evaluation of depressive symptoms combined with several of the most commonly used scales for the evaluation of manic symptoms [37]. The results of this study did not allow us to determine that this is the best option since mixed symptoms are not, from a phenomenological point of view, simply the coexistence of manic and depressive symptoms, but actually represent specific symptoms with differential characteristics. There are other studies in the literature that have tried to assess mixed symptoms. For example, the Koukopoulos Mixed Depression Rating Scale (KMDRS) [38] was validated to assessed mixed depression. Other studies have tried to validate scales intended to assess mixed states. However, to our knowledge, none have addressed manic and depressive symptoms while assessing mixed symptoms using a single assessment tool.

In short, the development of an instrument to assess the symptomatology present in bipolar disorder across the different episodes, of either polarity while including affective episodes that meet the criteria for a mixed symptom specifier, and even unipolar depression with mixed symptom specifier, is still unresolved. In view of the above, the present investigation has sought to:

1. Design a scale (Scale for the Assessment of Episodes in Bipolar Disorder, SAEBD) to assess the full spectrum of clinical manifestations present in bipolar disorder;
2. Analyze the ability of the scale to discriminate between the different clinical manifestations;
3. Analyze the diagnostic capacity of the scale (sensitivity, specificity, positive and negative likelihood value, positive and negative likelihood ratio) against other scales used to assess mood disorders.

## 2. Materials and Methods

### 2.1. Measures

#### 2.1.1. Clinical

A Clinical diagnosis was made in the inpatient unit or mental health center by the referring professional.

#### 2.1.2. Depressive Symptomatology

This was evaluated using two instruments: (a) the Hamilton Depression Scale, 17-item version (HAM-D): it is a hetero-administered scale, designed to measure the intensity and/or severity of depression. This scale is one of the most widely used to monitor the evolution of symptoms in clinical practice and research [29,30,31]. In the current study it is used to determine the presence or absence of depressive polarity. Scores equal to or less than 7 are considered compatible with euthymia. (b) Montgomery-Asberg Depression Scale (MADRS): A hetero-administered scale, consisting, in this case, of 10 items that evaluate the symptomatological profile and severity of depression [32,33]. In the current study it is used to determine the presence or absence of depressive polarity in a complementary way, as scores below 10 are compatible with euthymia.

#### 2.1.3. Manic Symptomatology

Young Mania Rating Scale (YMRS) This scale consists of 11 items hetero-administered [26,39]. In the present study, it was used to determine the presence or absence of manic polarity and scores equal to or less than 6 are compatible with euthymia.

### 2.2. Participants

Participants were included if they have a diagnosis of bipolar disorder. The euthymic subjects had to meet the criteria of scoring HAM-D < 8 and YMRS < 5. DSM-5 criteria were used in order to establish if the participants met the criteria for a depressive, hypomanic or manic episode. The mixed symptoms specifier was used to define significant mixed symptoms if at least three symptoms of the opposite polarity were present during the affective episode. Substance abuse, cognitive impairment, dementia, or any neurological disease affecting cognitive performance were exclusion criteria for participants.

### 2.3. Statistical Analysis

Statistical analyses were carried out using the Statistical Package for the Social Sciences (SPSS), version 23.0 for Windows (IBM Corp., Armonk, NY, USA). Exploratory factor analysis was carried out with the Jamovi v.1.6.23 computer software [40]. Descriptive and univariate statistics (chi-square) were used to compare the demographic and clinical characteristics between the subjects with type I and type II BD. Concerning content analysis of the items, inter-rater reliability was calculated with Krippendorff’s alpha statistic, in which values range from 0 to 1, where 0 is perfect disagreement and 1 is perfect agreement. Krippendorff [41] suggests requiring α ≥ 0.800 (2004, p. 241). Statistical analysis of the items required, firstly, the use of corrected item-total correlation, as this is one of the best item-assessment methods when constructing tests [42]. Secondly, reliability tests were used to check for internal consistency (Cronbach’s alpha, split-half), as well as the average interitem correlation, which is considered a much more useful index than coefficient alpha per se [43,44,45]. Sample sizes as small as 30 can measure alpha reliably so long as the scale items have strong inter-correlations (i.e., the average interitem correlations are in the 0.15–0.50 range) [43,44,45]. Then, as customary, the discriminative validity was assessed using the Mann Whitney U test to assess whether or not the items of depression and manic discriminate between subgroups (manic or depressive vs. euthymia). Likewise, a Kruskal-Wallis test was used to assess whether or not the items of general psychopatology discriminate among subgroups (manic, depressive, or mixed).

Next, construct validity, by means of factor analysis, was performed. Factor analysis with reduced sample sizes is dependent on the level of communalities of item variables [43,44,45]. To check this assumption, a principal component analysis was first performed; the eigenvalue for the first principal component was examined, as well as the loadings of the scale items on it. Then, an exploratory factor analysis was performed by the least residuals extraction method and Oblimin rotation. The number of factors was based on parallel analysis [46]. Then, the diagnostic ability of the scale, via discriminant analysis to predict group membership, was analyzed. Finally, the diagnostic test accuracy was checked using the area under the curve (AUC) and the ROC curves to calculate the sensitivity, specificity, positive and negative likelihood ratios [47].

### 2.4. Procedure

The patients were evaluated in the consultation rooms or in the short-term hospitalization unit (in case of admission). Before the evaluation they were informed of the present study and invited to participate. The evaluation was performed by a psychiatrist or a clinical psychologist.

The study was carried out in accordance with local regulations and the principles established internationally in the Declaration of Helsinki (last modification approved at the 52nd General Assembly, Edinburgh, Scotland; October 2000). Confidentiality was respected at all times (LOPD 15/99). The study was approved by the Ethics Committee of the hospital responsible for the study (Ethics Committee of Fundacion Investigacion Biomedica Hospital Ramon y Cajal (date of approval: 17 February 2017, project # PI16/01319).

## 3. Results

### 3.1. Sample

All subjects meeting the inclusion criteria were duly informed of the objectives and duration of the study and their participation was obtained by written informed consent.

In total, a sample of 88 subjects diagnosed with bipolar disorder, according to DSM-5 criteria, was collected over three years. These subjects were recruited from the patients attending the Hospital Universitario Ramón y Cajal (Madrid, Spain) and were evaluated by the psychiatrists in charge of the study. Of the total number of subjects, 67 (76.1%) met criteria for Bipolar Disorder Type I and 21 (23.9%) met criteria for Bipolar Disorder Type II according to DSM-5 criteria. At the time of data collection for the present study, 37 participants (42.0%) were in euthymia, 20 (22.7%) were mixed, 18 (20.5%) were depressed, and 13 (14.8%) were manic. The average number of years of evolution of the disorder was 22.2 years (standard deviation (sd) = 9.5; range: 3–52). The estimated average age of onset was 30.4 years (sd = 12.4; range: 3–52). The average number of hospitalizations was 4.3 (sd = 4.2, range: 0–18).

Of the total, 42 (47.7%) were men and 46 (52.3%) were women. The mean age was 51.2 years (sd = 13.4; range: 20–77). Additional information revealed that 22 (25%) live alone, 27 (30.7%) live with the family of origin, 39 (44.3%) live with their own family. Regarding marital status, 36.4% (*n* = 32) are single, 37.5% (*n* = 33) are married, 22.7% (*n* = 20) are separated or divorced and 2.3% are widowed. One person did not report his or her situation. Some 15.9% (*n* = 14) have primary education, 25% (*n* = 22) have secondary education and 59.12% (*n* = 52) have higher education. Regarding employment status, 29.5% (*n* = 26) are working, 27.3% (*n* = 24) are unemployed and 43.2% (*n* = 38) are retired or receiving a pension.

Regardless of the group to which the subjects belonged (euthymia, depression, mania or mixed), the participants were similarly distributed in terms of sex, cohabitation, marital status, educational level, and employment status (chi-square > 0.05 for all analyses). However, the estimated age of onset was earlier for the group with predominant manic symptoms and this group also required a greater number of hospitalizations. On the other hand, the group with BD-II diagnosis presented more years of evolution of the disorder and an earlier onset, but with fewer hospitalizations than the BD-I group.

### 3.2. Preliminary Analysis

The procedure involved the following phases:

1. The construction of a pool of 63 items. Their qualitative evaluation led to the elimination of 11 items considered redundant or inadequate. The remaining items were submitted to expert judges to assess their relevance and assignment to a category (depression, mania, etc.) for the population under study. This resulted in a scale with 15 items to assess manic polarity, 16 items to assess depressive polarity and 21 items to assess general psychopathology The concordance between the five judges who evaluated the 52 items in this phase was Krippendorff’s alpha (nominal data) = 0.89. The identification of unclear items and their improvement was a further step in the refinement of the scale.
2. Statistical analysis of the items. Using the items that passed the previous phase, we applied them to the sample under study. Once the data had been collected, homogeneity and internal consistency were analyzed in order to eliminate, if necessary, items that were not sufficiently consistent with the scale. In addition, the split-half procedure was used. The analyses indicated high reliability for the manic symptoms subscale (α = 0.95, split-half = 0.94), depressive (α = 0.98, split-half = 0.95), general psychopathology (α = 0.92, split-half = 0.88), as well as the scale considered globally (α = 0.96, split-half = 0.90). The average interitem correlation was 0.45.
3. Estimation of the discriminative capacity of the items of the subscales. Next, we determined the discriminant validity of each item of the instrument between subgroups in different states (euthymia, manic, depressive, mixed). A Mann-Whitney U test indicated that differences were statistically significant for all of the manic symptoms items and for all of the depressive symptoms items. A Kruskal-Wallis test revealed that nine items of the general psychopathology subscale did not differentiate among euthymia, mixed or manic subgroups, so they were removed from further analyses. With the remaining 43 items, we performed the exploratory factor analysis.
4. Exploratory factor analysis. To check if exploratory factor analysis could be used with reduced sample size, a principal component analysis (PCA) was carried out. The analysis revealed that the first eigenvalue (24.76), was higher than the required 6.00 value, so Exploratory Factor Analysis can be used. Once these criteria were verified, an exploratory factor analysis (EFA) was carried out. The analyses resulted in two factors. The first factor, with an eigenvalue of 20, explained 34.6% of the variance and measures depressive symptoms. The second factor, with an eigenvalue of 7.9, explained 33.2% of the variance and measures manic symptoms. Together, the two factors explained 67.8% of the variance. The correlation between both factors was 0.30. The measure, namely, the SAEBD (see Table A1 in Appendix A), was then utilized for further analyses, as explained below.

### 3.3. Discriminant Analysis

We then proceeded to perform a discriminant analysis in which we included all the items that were found to adequately discriminate in their respective diagnostic groups. The aim was to determine to what extent all the items of the scale allow us to distinguish between the four groups identified by their scores on the scales: euthymia, depressive, mixed, manic. Table 1 shows three discriminant functions. The first one, with an eigenvalue of 22.34, explains 63.6% of the variance; the second one, with an eigenvalue of 8.84, explains 26.2% of the variance and the third function, with an eigenvalue of 3.95, explains 11.3% of the variance. The three canonical correlations were high, indicating that the discriminant variables allow us to adequately differentiate between the groups.

Wilks’ lambda statistic expresses the proportion of variability not due to differences between groups. It tests the null hypothesis that the multivariate means of the groups (i.e., centroids) are equal. Values close to 1 indicate a strong resemblance between the groups, while values close to 0 indicate a strong difference between them. Table 2 shows the low lambda values and their proximity to values of 0, indicating an absence of overlap between the groups. This fact is further reinforced by the transformed value of lambda (chi-square), which is significant in all three cases, allowing us to reject the null hypothesis that the groups compared have equal means in the discriminant variables.

The structure matrix (see Table 3) shows the variables ordered by their degree of correlation (from highest to lowest) with the discriminant function. The first discriminant function groups items related fundamentally to the depressive category; the second function groups items related to the manic category, and the third function only includes one item that correlates with the other functions as well.

The values of the centroids of each group (euthymia, depressive, mixed, manic) in each function (see Table 4), allow us to note how the group in euthymia is characterized by low scores in all three functions and especially in the first function which, as we pointed out, indicates higher depressive symptoms. Consistent with these results, those in the depressive group obtained high scores in the first function, followed by low scores in the second function (denoting manic symptoms) and low scores, although to a lesser extent, in the third function (general psychopathology). Meanwhile, the group within the manic group obtained low scores on the first function (depressive), high scores on the second function (manic), followed by low scores on the third function (general psychopathology). Finally, the mixed symptoms group obtained high scores, especially on the second function, followed by high scores on the first function, but less extreme than the groups with a clear affective episode (either depressive or manic), and high scores on the third function (general psychopathology).

**Figure 1:**
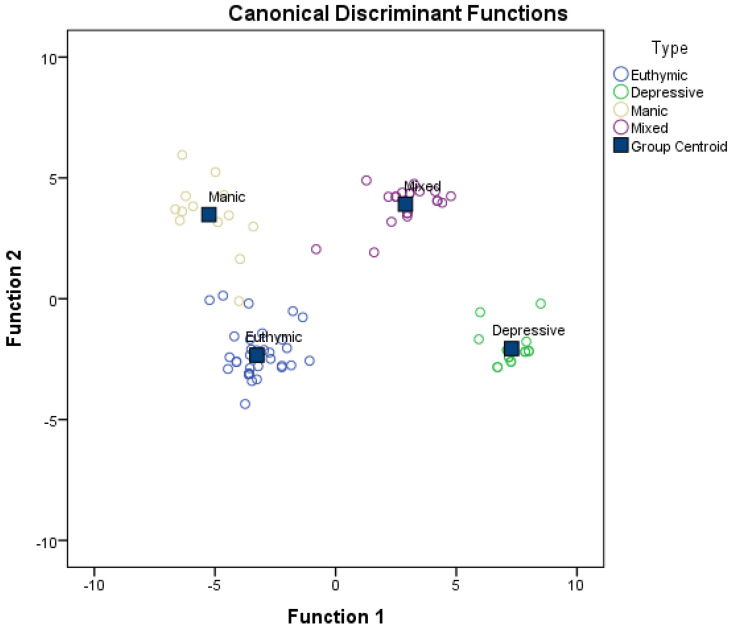
Scatterplot for the four groups.

Figure 1 shows the scatter plot of all the cases used in the analysis on the plane defined by the first two discriminant functions. The absence of overlap among the groups and the usefulness of the two functions for classifying the groups can be seen.

The confusion matrix (see Table 5) shows how the discriminant functions correctly classify 100% of the cases in euthymia, depressive or mixed categories, as well as 92.3% of the cases with predominant manic symptoms. Overall, the functions correctly classify 98.9% of the cases.

The results obtained indicate that the selected items adequately discriminate between the different diagnostic subgroups, which confirm the discriminant validity of the scale.

### 3.4. Diagnostic Capacity of the Scale

We then calculated the diagnostic capacity of the SAEBD, taking into account the diagnosis of the current state of the subjects (clinical cases or not -in euthymia-) and the total of the SAEBD, with the items retained after eliminating those without sufficient discriminative power. To this end, a sample of 60 positive (i.e., clinical) and 28 negative (i.e., in euthymia) cases, as determined by the DSM5 structured interview done by the clinical team, were used. In addition, the diagnostic capacity of the SAEBD was compared with that of the scales also applied in the present study.

To interpret the results, the area under the curve (AUC) has a value between 0.5 and 1, where 1 represents a perfect diagnostic value and 0.5 is a test without diagnostic discriminatory capacity. That is, if the AUC for a diagnostic test is 0.8, there is an 80% probability that the diagnosis made on a patient is more correct than that of a healthy person chosen at random. Thus, the diagnostic test with the highest area under the curve is always chosen. As for ROC curves, the following intervals have been established for AUC values: A bad test has values between 0.5 and 0.6, a fair test between 0.6 and 0.75, a good test between 0.75 and 0.9 and values between 0.97 and 1 means it is an excellent test.

Table 6 shows the results after comparing the curves of the different instruments used. The superior capacity of the SAEBD can be seen, with a value of the area under the curve (0.935) which denotes a very high capacity of the scale to discriminate between clinical and non-clinical cases (i.e., in euthymia). That is, there is a 93.5% probability that, when faced with a pair of persons, one clinical and the other non-clinical, the test will classify them correctly. The confidence interval (0.88 to 0.099) is equally satisfactory. The cut-off point that maximizes sensitivity and specificity is 6. Although the results of the other scales were also satisfactory, those obtained with the SAEBD were relatively superior.

Based on the cut-off point established for the SAEBD, and after recoding the scores of the participants as less than or greater than 6 points, the sensitivity, specificity, positive and negative likelihood ratios were calculated, contrasting it with the gold standard, which is the state of euthymia or not assessed by the medical staff. Table 7 shows the number of subjects with and without the disease who test positive and negative for each of the measures. For the SAEBD, the test sensitivity was 0.95 [95% CI = 0.85, 0.99], test specificity was 0.71 [95% CI = 0.51, 0.86], the Positive Predictive Value (PPV) was 0.88 [95% CI = 0.77, 0.94], the Negative Predictive Value (NPV) was 0.87 [95% CI = 0.65, 0.97] the Positive Likelihood Ratio (+LR) was 3.33 [95% CI: 1.85, 0.99], and the Negative Likelihood Ratio (–LR) was 0.07 [95% CI = 0.02, 0.22]. The same procedure was carried out with the other scales used in the present study for the evaluation of depression and mania, using, in each case, the cut-off points established in the scales and comparing it with the gold standard mentioned above. For the MADRS, the test sensitivity was 0.53 [95% CI = 0.40, 0.66], test specificity was 1.00, [95% CI = 0.85, 1.00], the PPV = 1.00 [95% CI = 0.87, 1.00], the NPV = 0.50 [95% CI = 0.37, 0.63] the +LR was infinite [95% CI: 1.96, 487], and the –LR was 0.47 [95% CI = 0.36, 0.62]. For the HAM-D, the test sensitivity was 0.62 [95% CI: 0.48, 0.74], test specificity was 1.00 [95% CI = 0.88, 1.00], the PPV = 1.00 [95% CI = 0.88, 1.00], the NPV = 0.55 [95% CI = 0.40, 0.69], the +LR was infinite [95% CI: 2.27, 561], and the –LR was 0.38 [95% CI = 0.28, 0.54]. For the YMRS, the test sensitivity was 0.65 [95% CI = 0.51, 0.77], test specificity was 0.89 [95% CI = 0.71, 0.97], the PPV = 0.93 [95% CI = 0.79, 0.98], the NPV = 0.54 [95% CI = 0.39, 0.69], the +LR was 6.07 [95% CI: 2.05, 18], and the –LR was 0.39 [95% CI = 0.27, 0.57].

## 4. Discussion

The SAEBD scale has a high discriminatory capacity. It has two main functions, the first groups items related to depressive symptoms and the second groups items related to manic symptoms. These functions allow it to adequately classify the different groups evaluated without overlapping. The discriminant functions allow the correct classification of 100% of the cases in euthymia, with a depressive episode or with mixed symptoms, as well as 92.3% of the cases with a manic episode. Overall, the functions correctly classified 98.9% of the cases. The classification is in accordance with expectations, which gives the scale construct validity.

The SAEBD area under the curve (0.935) denotes a very high capacity to discriminate between clinically active and non-clinically active cases (i.e., in euthymia). The value is higher than that obtained with other instruments using the same sample and which generally assess states compatible with euthymia or clinical. Furthermore, when the diagnostic capacity of the SAEBD is analyzed, taking into account a medical diagnosis of euthymia or non-euthymia according to DSM criteria by a medical team outside the study using a cut-off of 6 points, the scale shows a sensitivity of 0.95 and a specificity of 0.71, a Positive Predictive Value of 0.88 and a Negative Predictive Value of 0.87. The values obtained are higher than those obtained in other investigations using clinical markers [48,49] or other instruments commonly used [50,51,52,53,54,55,56], or recently designed [57,58] for the evaluation of mood disorders. The SAEBD yields diagnostic sensitivity and specificity results comparable to other studies carried out on a larger scale with a Spanish-speaking population [59].

Specifically, the predictive values of the SAEBD indicate that there is a 77% to 95% probability of having a diagnosis of bipolar disorder with a positive test result, and a 65% to 97% probability of not having the diagnostic characteristics with a negative test result. This also means that between 23% and 35% of patients will not be identified or will be identified incorrectly. Hence the importance of taking into account that this scale has a screening character that allows it to be used in primary care. Screening tools could be very helpful for primary healthcare clinicians as they usually lack enough time or training to perform more thorough examinations but have more frequent access to patients and they are usually the first contact for patients with the healthcare system when in distress. Screening tools aim to identify patients’ needs early enough to provide adequate care and reduce healthcare costs [60]. Considering the frequent long delay in diagnosis in bipolar disorder [7], an early indication of active mood symptoms could help clinicians to be aware on how to appropriately refer to specialist services or on how to monitor them to detect early signs of mood swings when they are already diagnosed but currently monitored also by primary healthcare [24]. However, this does not eliminate the need to carry out a complete clinical evaluation by the competent specialist team.

Regarding other potential implications, the use of this screening tool could allow clinicians to assess manic, depressive, and mixed symptoms in a structured way and timely monitoring framework. It could help clinicians to overcome typical confusion around the DSM-5 specifier, providing a systematic approach in assessing mood symptoms.

The study was conducted in a clinical context, although subjects in different states have been included in order to broaden the generalizability of the results. Several limitations can be noted. Firstly, the sample, although comparable in size to other studies, is relatively small, so it is advisable to extend the study to a more diverse population with bipolar disorder, both in sociodemographic and clinical characteristics. Secondly, administering the scale would require that clinicians are trained in assessing mood symptoms (which General Practitioners are trained to some extent) but would not be appropriate for other roles. Also, it would not be appropriate as a self-administered tool, as it relies on the availability of a clinician to administer it. Furthermore, despite the aim of being as short as possible in order to maintain acceptable reliability and validity, it will require a few minutes to administer the relevant questions needed to cover all the items.

In the present study, we compared the diagnostic capacity of the SAEBD with other instruments for the assessment of states compatible with depressive, manic, or mixed symptoms. Additionally, if scores were obtained in both subscales, it suggests significant mixed symptoms, which is in line with the use of the DSM-5 mixed specifier. In future studies it would be advisable to compare the diagnostic accuracy of this screening tool with other existing ones. Also, reliable shorter scales could be a relevant direction in order to keep efficiency in assessments. Moreover, studies using this scale as part of treatment monitoring could allow clinicians to have a more objective follow-up measure that may help to inform therapeutic decisions.

## 5. Conclusions

The SAEBD is a reliable and valid instrument, which can distinguish between subjects with predominant manic, depressive, or mixed symptoms, or euthymic subjects.

The SAEBD shows levels of sensitivity and specificity comparable or superior to other existing instruments in the clinical and research context.

Since the validation of a scale is an ongoing process, it is necessary to continue research on the properties of the scale and to compare its properties with those of other instruments designed for the assessment of bipolar disorder.

## Figures and Tables

**Table 1:** Eigenvalues of discriminant functions.

Function	Eigenvalue	% of Variance	% Cumulative	Canonical Correlation
1	22.335	63.6	63.6	0.978
2	8.836	26.2	88.7	0.948
3	3.952	11.3	100.0	0.893

**Table 2:** Wilks’ lambda of discriminant functions.

Function Test	Wilks’ Lambda	chi-square	DF	*p*
1–3	0.001	446.777	129	<0.001
2–3	0.021	246.755	84	<0.001
3	0.202	101.591	41	<0.001

DF, Degrees of Freedom.

**Table 3:** Structure matrix.

Items	Function			Items	Function		
	1	2	3		1	2	3
D1	0.240 *	0.014	−0.049	G9	0.078	0.376 *	0.236
D13	0.210 *	0.049	−0.031	M12	0.034	0.372 *	0.162
G1	0.208 *	0.193	0.149	M5	0.014	0.351 *	0.070
D2	0.205 *	0.023	−0.047	M4	0.013	0.345 *	0.081
D5	0.205 *	−0.021	−0.072	M9	0.069	0.334 *	0.188
D4	0.204 *	0.019	−0.052	M8	0.025	0.330 *	0.059
D3	0.204 *	0.026	−0.049	M10	0.031	0.329 *	0.074
D6	0.162 *	0.078	0.040	M7	0.004	0.324 *	0.104
D9	0.144 *	0.023	0.023	G17	0.149	0.283 *	0.026
G5	0.138 *	0.134	0.027	M15	0.016	0.276 *	0.111
D8	0.133 *	0.054	0.066	G18	0.114	0.238 *	0.184
D7	0.131 *	0.055	0.027	M6	0.050	0.233 *	0.127
D15	0.128 *	0.037	0.016	G13	0.038	0.202 *	0.046
D14	0.126 *	0.080	0.069	G8	0.031	0.194 *	0.132
D10	0.117 *	0.077	0.063	G14	0.005	0.184 *	0.035
D11	0.108 *	0.090	0.084	M11	0.040	0.175 *	0.146
D16	0.088 *	0.027	0.050	G12	0.053	0.173 *	0.147
M13	0.050	0.469 *	0.149	G19	0.072	0.168 *	0.071
M2	0.046	0.438 *	0.153	G15	0.050	0.168 *	0.157
M3	0.100	0.426 *	0.313	G16	0.052	0.165 *	0.028
M1	0.008	0.395 *	0.037	D12	0.076	0.090	0.099 *
M14	0.066	0.380 *	0.145				

Note: Pooled within-groups correlations between discriminating variables and standardized canonical discriminant functions. Variables ordered by absolute size of correlation within function. * Largest absolute correlation between each variable and any discriminant function.

**Table 4:** Values of the centroids for each group in the discriminant functions.

Type	Function		
	1	2	3
Euthymic	−3.265	−2.335	0.936
Depressive	7.289	−2.064	−1.834
Manic	−5.249	3.481	−3.386
Mixed	2.881	3.914	2.121

**Table 5:** Classification results.

Group Membership	Type	Predicted Group Membership			
		Euthymia	Depression	Mania	Mixed
Original	Euthymia	37 (100.0%)	0	0	0
	Depression	0	18 (100.0%)	0	0
	Mania	1 (7.7%)	0	12 (92.3%)	0
	Mixed	0	0	0	20 (100.0%)

98.9% of original cases correctly classified.

**Table 6:** Areas under the curve of the instruments used.

Test Result Variables	Area	Standard Error	Asymptotic Significance	95% Asymptotic Confidence Interval	
				Lower Bound	Upper Bound
SAEBD	0.935	0.027	<0.001	0.882	0.987
MADRS	0.891	0.034	<0.001	0.825	0.958
HAM-D	0.867	0.037	<0.001	0.793	0.940
YMRS	0.809	0.046	<0.001	0.719	0.898

SAEBD, Scale for the Assessment of Episodes in Bipolar Disorder; MADRS, Montgomery-Asberg Depression Scale; HAM-D, Hamilton Depression Scale; YMRS, Young Mania Rating Scale.

**Table 7:** Numbers of subjects with and without the disorder who test positive and negative in the scales.

Scale Result	Disorder Present	Disorder Absent	Total
SAEBD			
Test positive	57	8	65
Test negative	3	20	23
Total	60	28	88
MADRS			
Test positive	32	0	32
Test negative	28	28	56
Total	60	28	88
HAM-D			
Test positive	37	0	37
Test negative	23	28	51
Total	60	28	88
YMRS			
Test positive	39	3	42
Test negative	21	25	46
Total	60	28	88

## Data Availability

The data that support the findings of this study are available from the corresponding author, upon reasonable request.

## Appendix A

**Table A1:** The SAEBD (Scale for the Assessment of Episodes in Bipolar Disorder).

**Manic Symptoms Subscale (MSS)**	**Scores**			
01. Euphoria	0	1	2	3
02. Increased goal-directed activity	0	1	2	3
03. Distraction	0	1	2	3
04. Involvement in risky activities	0	1	2	3
05. Increased self-esteem	0	1	2	3
06. Aggressiveness	0	1	2	3
07. Verbosity	0	1	2	3
08. Tachypsychia	0	1	2	3
09. Flight of ideas	0	1	2	3
10. Grandiose delusions	0	1	2	3
11. Extravagant appearance	0	1	2	3
12. Expansive contact	0	1	2	3
13. Diminished need for sleep	0	1	2	3
14. Global insomnia, without associated tiredness	0	1	2	3
15. Increased sexual interest	0	1	2	3
16. Catastrophic ideas of ruin	0	1	2	3
17. Irritability	0	1	2	3
18. Psychomotor agitation	0	1	2	3
19. Auditory accusatory or denunciatory hallucinations	0	1	2	3
20. Delusional ideas of harm	0	1	2	3
21. Delusional ideas of reference	0	1	2	3
22. Confusional symptoms	0	1	2	3
23. Unkempt appearance, neglect of personal cleanliness	0	1	2	3
24. Conciliation insomnia	0	1	2	3
25. Maintenance insomnia	0	1	2	3
26. Late insomnia (early awakening)	0	1	2	3
**Total MSS:**				
**Depressive Symptoms Subscale (DSS)**	**Scores**			
01. Depressed mood	0	1	2	3
02. Decreased interest	0	1	2	3
03. Decreased enjoyment	0	1	2	3
04. Abulia	0	1	2	3
05. Hypoergia	0	1	2	3
06. Passive thoughts of death	0	1	2	3
07. Bradypsychia	0	1	2	3
08. Negative self-concept	0	1	2	3
09. Low self-esteem	0	1	2	3
10. Hypochondriasis	0	1	2	3
11. Delusional ideas of guilt	0	1	2	3
12. Dejected contact	0	1	2	3
13. Decreased appetite	0	1	2	3
14. Decreased sexual interest	0	1	2	3
15. Hypersomnia	0	1	2	3
16. Concentration difficulties	0	1	2	3
17. Internal tension	0	1	2	3
**Total DSS:**				
**TOTAL SAEBD:**				

Scores: 0 = absent, 1 = mild, 2 = moderate, 3 = severe. Interpretation: (a) Scores above 6 points on the total scale indicate clinical status. (b) If scores are obtained in only one of the subscales, it is indicative of predominant manic symptoms or depressive symptoms, respectively. (c) If scores are obtained in both subscales, it suggests significant mixed symptoms. (d) A state of euthymia requires not exceeding the cut-off point of 6 points on the scale.
